# Saikosaponin-d, a calcium mobilizing agent, sensitizes chemoresistant ovarian cancer cells to cisplatin-induced apoptosis by facilitating mitochondrial fission and G2/M arrest

**DOI:** 10.18632/oncotarget.21076

**Published:** 2017-09-16

**Authors:** Hideaki Tsuyoshi, Vincent Kam Wai Wong, Yu Han, Makoto Orisaka, Yoshio Yoshida, Benjamin K. Tsang

**Affiliations:** ^1^ Department of Obstetrics & Gynecology, Cellular & Molecular Medicine, Interdisciplinary School of Health Sciences, University of Ottawa, Ottawa, Ontario, Canada; ^2^ State Key Laboratory of Quality Research in Chinese Medicine, Macau Institute for Applied Research in Medicine and Health, Macau University of Science and Technology, Avenida Wai Long, Taipa, Macau, China; ^3^ Chronic Disease Program, Ottawa Hospital Research Institute, Ottawa, Ontario, Canada; ^4^ Department of Obstetrics & Gynecology, University of Fukui, Fukui, Japan

**Keywords:** ovarian cancer, chemoresistance, mitochondrial dynamics, G2/M arrest, Ssd

## Abstract

Cisplatin (CDDP) and its derivatives are first line anti-cancer drugs for ovarian cancer (OVCA). However, chemoresistance due to high incidence of p53 mutations leads to poor clinical prognosis. Saikosaponin-d (Ssd), a saponin from a herbal plant extract, has been shown to induce cell death and sensitize chemoresistant cells to chemotherapeutic agents. Here, we demonstrated that Ssd sensitized chemoresistant OVCA cells with either p53-wt, -mutant and -null to CDDP. The action of Ssd appears to be through induction of mitochondrial fragmentation and G2/M arrest. Ssd is mediated via calcium signaling, up-regulation of the mitochondrial fission proteins Dynamin-related protein 1 (Drp1) and optic atrophy 1 (Opa1), and loss in mitochondrial membrane potential (MMP). Moreover, in the presence of CDDP, Ssd also down-regulates protein phosphatase magnesium-dependent 1 D (PPM1D) and increases the phosphorylation of checkpoint protein kinases (Chk) 1, cell division cycle 25c (Cdc25c) and Cyclin dependent kinase 1 (Cdk1). Our findings suggest that Ssd could sensitize OVCA to CDDP independent of the p53 status through multiple signaling pathways. They support the notion that Ssd may be a novel adjuvant for the treatment of chemoresistant OVCA.

## INTRODUCTION

Ovarian cancer (OVCA) is the most lethal gynecological malignancy and ranks fifth in cancer deaths among women [1]. Cisplatin (CDDP) and its derivatives are first line chemotherapeutic agents for OVCA. Resistance to chemotherapy remains a major hurdle to successful treatment and is associated with poor prognosis. p53 is believed to be a central transcription factor in the molecular mechanisms underlying CDDP sensitivity and a high incidence of p53 mutation is evident in high grade serous OVCA [2].

Saikosaponin-d (Ssd), a major triterpenoid saponin derived from Bupleurum falcatum L. (Umbelliferae), is commonly used against inflammatory and infectious diseases. Ssd can exhibit anti-cancer activities [3, 4], which is mediated by the induction of apoptosis and autophagic cell death regardless of p53 status [5–7]. Moreover, low dose Ssd is capable of sensitizing chremosensitive or chemoresistant lung and cervical cancer or hepatoma cells to anti-cancer drug- or radiation-induced apoptosis [6, 8], suggesting that Ssd could have a potential to overcome the chemoresistance in OVCA cells, although the precise mechanisms of action of Ssd remain unknown.

Mitochondria are highly dynamic organelles, and their fission and fusion fulfill mitochondrial function, including respiration, calcium buffering, apoptosis, and autophagy [9]. Dynamin-related protein 1 (Drp1) controls the balance between fission and fusion by the phosphorylation at two distinct serine moieties. Phosphorylation of Ser616 activates Drp1 and induces mitochondrial fission mediated by Cyclin-dependent kinase 1 (Cdk1)/Cyclin B1 during mitosis [10]. In contrast, Drp1 is inactivated via Ser637 phosphorylation by protein kinase A, resulting in mitochondrial fusion while its dephosphorylation by the calcium-sensitive protein phosphatase calcineurin leads to mitochondrial fission [11, 12]. Drp1 may also be up-regulated by calcium/calmodulin–dependent protein kinase I (CaMKI) following intracellular calcium signaling, although the mechanism involved is not known [13, 14]. Mitochondrial fusion may also be induced by mitofusin-1 and mitofusin-2 at the outer mitochondrial membrane and by optic atrophy 1 (Opa1) [15]. Although mitochondrial dynamics can be relevant to cancer growth or death, limited evidence exists supporting its importance in the control of chemosensitivity in OVCA.

Cdk1 is maintained in an inactive state by phosphorylation of Thr14 and Tyr15, and is activated through dephosphorylation by cell division cycle 25c (Cdc25). Upon activation, it associates with Cyclin B1 in cytoplasm and translocates into the nucleus, where it promotes G2/M transition. DNA damage activates checkpoints to delay cell cycle progression. p53 up-regulates G1/S transition involving in CDDP-induced apoptosis, while Checkpoint protein kinase 1 (Chk1) prevents the entry into M phase of cells with DNA damage [16]. DNA damage activates Chk1, which phosphorylates Cdc25c, leading to the inhibition of Cdk1/Cyclin B1 interaction and resulting in G2/M arrest. However, protein phosphatase magnesium-dependent 1D (PPM1D) directly inhibits the activation of Chk1 in CDDP-induced Ser345 phosphorylation and confers chemoresistance in OVCA cells [17, 18].

In the present study, we have examined the hypothesis that Ssd sensitizes chemoresistant OVCA cells to CDDP-induced apoptosis by facilitating mitochondrial fission and G2/M cell cycle arrest. In studying the mechanism of action of Ssd, we have demonstrated that Ssd increases cytosolic calcium ions (Ca^2+^) concentration ([Ca^2+^]c) and facilitates CDDP-induced mitochondrial membrane potential (MMP) loss and mitochondrial fission in chemoresistant cells. Ssd also down-regulates PPM1D content, leading to the activation of Chk1, phosphorylation of Cdc25c and Cdk1, and subsequent G2/M arrest and apoptosis. Our data identify Ssd as a potentially novel therapeutic agent for chemoresistant human OVCA.

## RESULTS

### Ssd induces apoptosis and sensitizes OVCA cells to CDDP in p53-independent manner

**Figure 1:**
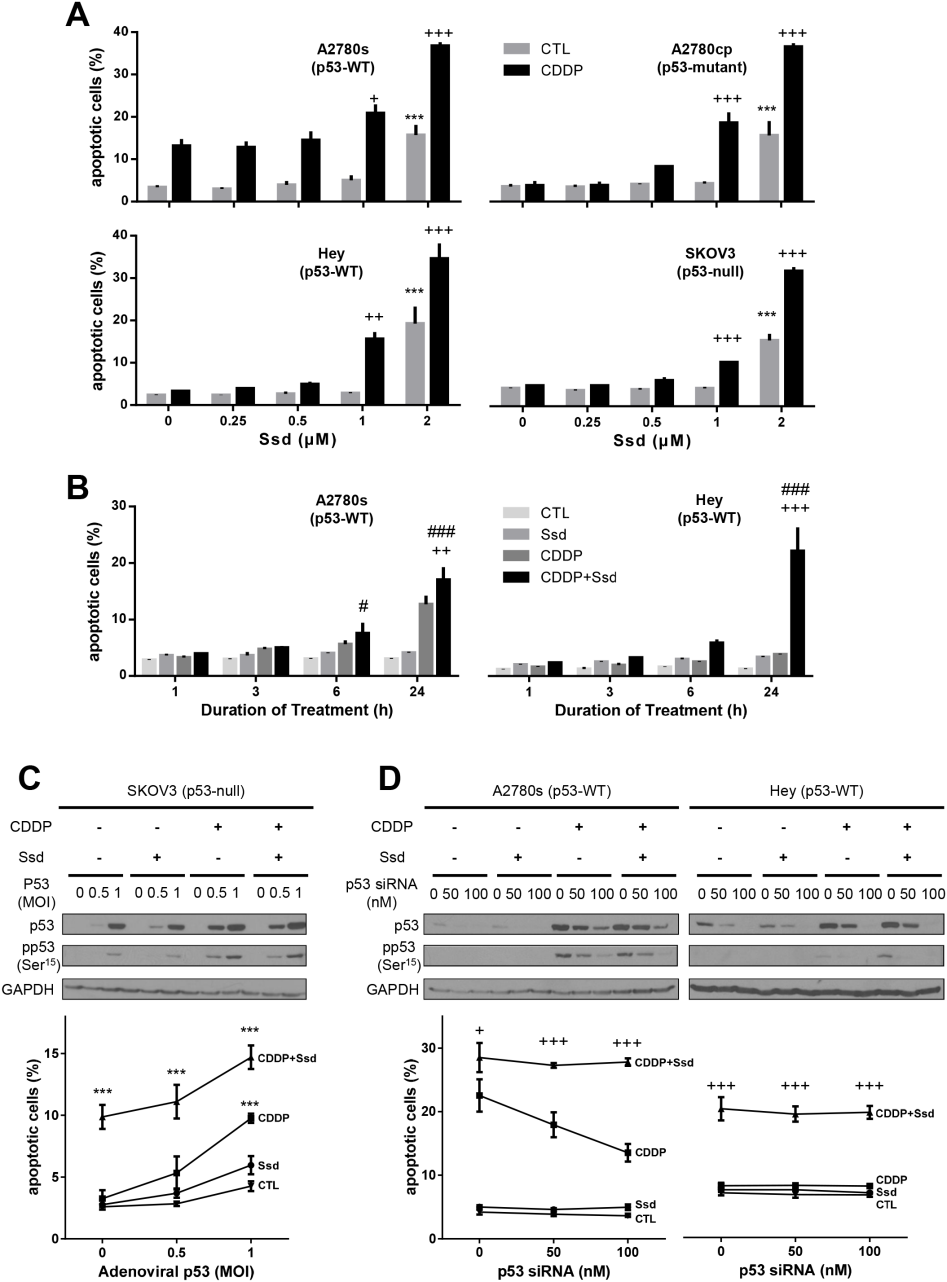
Ssd-induced apoptosis and sensitization of OVCA cells to CDDP in p53-independent manner **(A)** Ssd increased apoptosis and in the presence of CDDP Ssd sensitized OVCA cells to CDDP regardless of p53 status. A2780s, A2780cp, Hey and SKOV3 cells were cultured with CDDP (10 μM) and/or Ssd (0-2 μM, 24 h) and apoptosis were assessed. **(B)** Ssd-induced sensitization was time-dependent. A2780s and Hey cells were cultured with CDDP (10 μM) and/or Ssd (1 μM, 0-24 h). **(C)** The infection with adenoviral p53 had no significant affect on Ssd-sensitized apoptosis. SKOV3 cells were infected with adenoviral p53 (MOI = 0–1; 4 h), cultured with CDDP (10 μM) and/or Ssd (1 μM, 24 h) and apoptosis were assessed. p53, anti-phospho-Ser^15^-p53 and GAPDH contents were assessed by WB. **(D)** The treatment with p53 siRNA failed to decrease Ssd-sensitized apoptosis. A2780s and Hey cells were treated with p53 siRNA (0–100 nM, 12 h), cultured with CDDP (10 μM) and/or Ssd (1 μM, 24 h). ^***^P<0.001 (vs respective CTL), ^+^*P*<0.05, ^++^*P*<0.01 and ^+++^*P*<0.001 (vs respective CDDP) and ^#^*P*<0.05 and ^###^*P*<0.001 (vs respective Ssd). (*n*=3).

We firstly examined whether Ssd induces apoptosis and sensitize OVCA cells to CDDP by using chemosensitive A2780s cells and chemoresistant cells with different p53-status [p53-mutant (A2780cp), wild type (wt)-p53 (Hey), and p53-null (SKOV3)]. In chemosensitive cells (A2780s), CDDP alone (10μM; 24h) significantly induced apoptosis while exhibiting no significant effects in chemoresistant cells (A2780cp, Hey and SKOV3). Ssd alone (2μM; 24h) significantly increased apoptosis in all OVCA cells studied. While a lower concentration (1μM) was ineffective alone, Ssd sensitized these OVCA cells to CDDP-induced apoptosis regardless of their p53 status (Figure 1A). This response was concentration- and time-dependent (Supplementary Figure 1A & 1B). Reconstitution of p53-null SKOV3 with wt-p53 significantly facilitated CDDP-induced apoptosis, suggesting p53 could be essential to CDDP-induced apoptosis. However, this response was not influenced by the presence of Ssd (Figure 1C). In addition, silencing p53 by siRNA significantly decreased CDDP-induced apoptosis in wt-p53 A2780s (chemosensitive). However, in the presence of Ssd, this action was not effective in both chemosensitive and chemoresistant cells (Figure 1D), supporting the notion that p53 is not involved in Ssd-induced sensitization of OVCA cells.

We also investigated whether Ssd induces apoptosis and sensitizes other cancer cells to CDDP. The IC_50_ of CDDP alone for chemoresistant and chemosensitive SGC-7901 (gastric cancer) were 9.13 and 1.64μM, respectively while Ssd has no influence (Supplementary Figure 1B). In the present of 2.5 and 5μM Ssd, IC_50_ of CDDP for the chemoresistant cells was 4.25 and 2.50μM, respectively. Collectively, these findings suggest that Ssd sensitizes chemoresistant cancer cells to CDDP in a p53-independent manner.

### Ssd induces the mitochondrial fragmentation via inhibition of phospho-Ser^637^-Drp1 and activation of Opa1 processing in Oma1 and p53-independent manner

**Figure 2:**
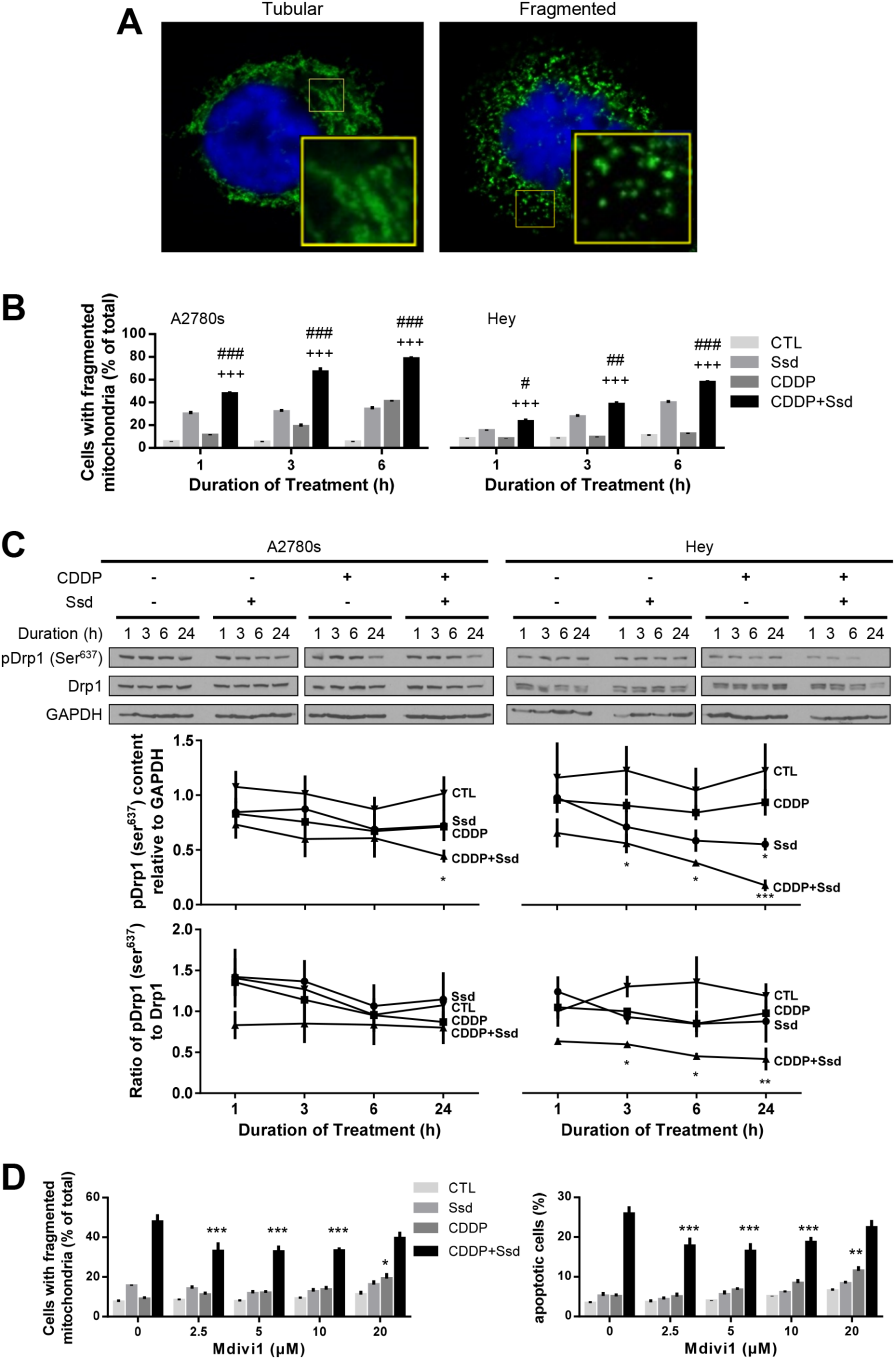
Ssd-induced mitochondria fragmentation via decrease of phospho-Ser637-Drp1 in chemoresistant OVCA cells **(A)** OVCA cells showed two major mitochondrial phenotypes: tubular and fragmented. The inset is an enlarged image of box area. **(B)** Ssd induced mitochondrial fragmentation and sensitized chemoresistant OVCA cells to CDDP. A2780s and Hey cells were cultured with CDDP (10 μM) and/or Ssd (1 μM, 0-6 h) and mitochondrial phenotype was assessed (*n*=3). ^#^*P*<0.05, ^##^*P*<0.01 and ^###^*P*<0.001 (vs respective Ssd) and ^+++^*P*<0.001 (vs respective CDDP). **(C)** Ssd decreased phospho-Ser^637^-Drp1 content and the combination with CDDP decreased phospho-Ser^637^-Drp1 content and the ratio of phospho-Ser^637^-Drp1 to Drp1 more than Ssd alone. A2780s and Hey cells were cultured with CDDP (10 μM) and/or Ssd (1 μM, 0-24 h). Drp1, phospho-Ser^637^-Drp1 and GAPDH contents were assessed by WB (*n*=3).^*^P<0.05, ^**^P<0.01 and^***^P<0.001 (vs respective CTL). **(D)** Ssd-induced mitochondrial fragmentation and apoptosis were attenuated by mdivi1. Hey cells were cultured with CDDP (10 μM), Ssd (1 μM) and mdivi1 (0-20 μM, 24 h). Mitochondrial phenotype and apoptosis were assessed (*n*=3). ^*^P<0.05, ^**^P<0.01 and ^***^P<0.001 (vs respective mdivi1 = 0 μM).

To determine if and how mitochondrial dynamics are involved in the regulation of chemosensitivity in OVCA cells, tubular and fragmented mitochondrial morphology was defined in both chemosensitive (A2780s) and chemoresistant (Hey) cells, in the absence of CDDP and Ssd treatment (Figure 2A). CDDP alone significantly increased the number of fragmented mitochondria in only the chemosensitive cells and in a time-dependent manner, while Ssd alone significantly induced this response in both chemosensitive and chemoresistant cells. Moreover, the number of both chemosensitive and chemoresistant cells with fragmented mitochondria were significantly higher in the presence of both Ssd and CDDP than of either agent alone (Figure 2B). Western blot (WB) analysis indicated that CDDP alone failed to alter phospho-Ser^637^-Drp1 content in both cell lines, suggesting phospho-Ser^637^-Drp1 may not be involved CDDP-induced mitochondrial fragmentation in chemosensitive cells. However, Ssd alone decreased phospho-Ser^637^-Drp1 content in chemoresistant cells. Moreover, phospho-Ser^637^-Drp1 content and the ratio of phospho-Ser^637^-Drp1 to Drp1 were down-regulated to a greater extent by Ssd in the presence of CDDP, suggesting Ssd-induced mitochondrial fragmentation may be mediated via decreased phospho-Ser^637^-Drp1 and the presence of CDDP sensitized Ssd-effect in chemoresistant cells (Figure 2C). Importantly, Ssd-induced mitochondrial fragmentation and apoptosis were attenuated by the Drp1 inhibitor mitochondrial division inhibitor-1 (mdivi-1) (Figure 2D), suggesting that Drp1 may be a pivotal participant in the Ssd-induced fragmentation and apoptosis in chemoresistant cells.

Our previous studies have shown the proteolytic activation of the mitochondrial fusion protein Opa1 by Oma1 (evident by 2 intact bands and 3 cleaved products on WB) results in mitochondrial fragmentation and is also a determinant of CDDP sensitivity in OVCA [15]. We have examined the influence of CDDP, alone or in combination with Ssd, on the ratio of short form to long form as an indication of Opa1 activation. CDDP (alone or with Ssd) increased this ratio in chemosensitive cells, but Ssd alone was ineffective, suggesting CDDP-induced mitochondrial fragmentation in chemosensitive cells may be mediated via Opa1 processing. In contrast, while CDDP alone appeared to have no effect on the ratio of the Opa1 isoform in chemoresistant cells, its action was facilitated by the presence of Ssd, suggesting that Ssd may sensitize CDDP-induced Opa1 processing in chemoresistant cells. We have previously shown that CDDP induces the p53-depending activation of Oma1 (40kDa) and subsequent mitochondrial fragmentation in chemosensitive but not chemoresistant OVCA cells [15]. As expected, CDDP alone increased the Oma1 content in chemosensitive but not chemoresistant cells (Supplementary Figure 2). Oma1 activation was not observed in the presence of Ssd irrespective of the presence of CDDP, suggesting this combination induces Opa1 processing directly or via pathways independent of Oma1 and p53. Collectively, our findings suggest mitochondrial fragmentation in chemosensitive cells is mainly induced by CDDP via Oma1 activation/Opa1 processing in a p53-dependent manner while decreased phospho-Ser^637^-Drp1 is mainly involved in Ssd-induced mitochondrial fragmentation and the sensitization of chemoresistant cells to CDDP.

### Ssd increases [Ca^2+^]c and, in combination with CDDP, induces MMP loss and activates CaMKI in OVCA cells

**Figure 3:**
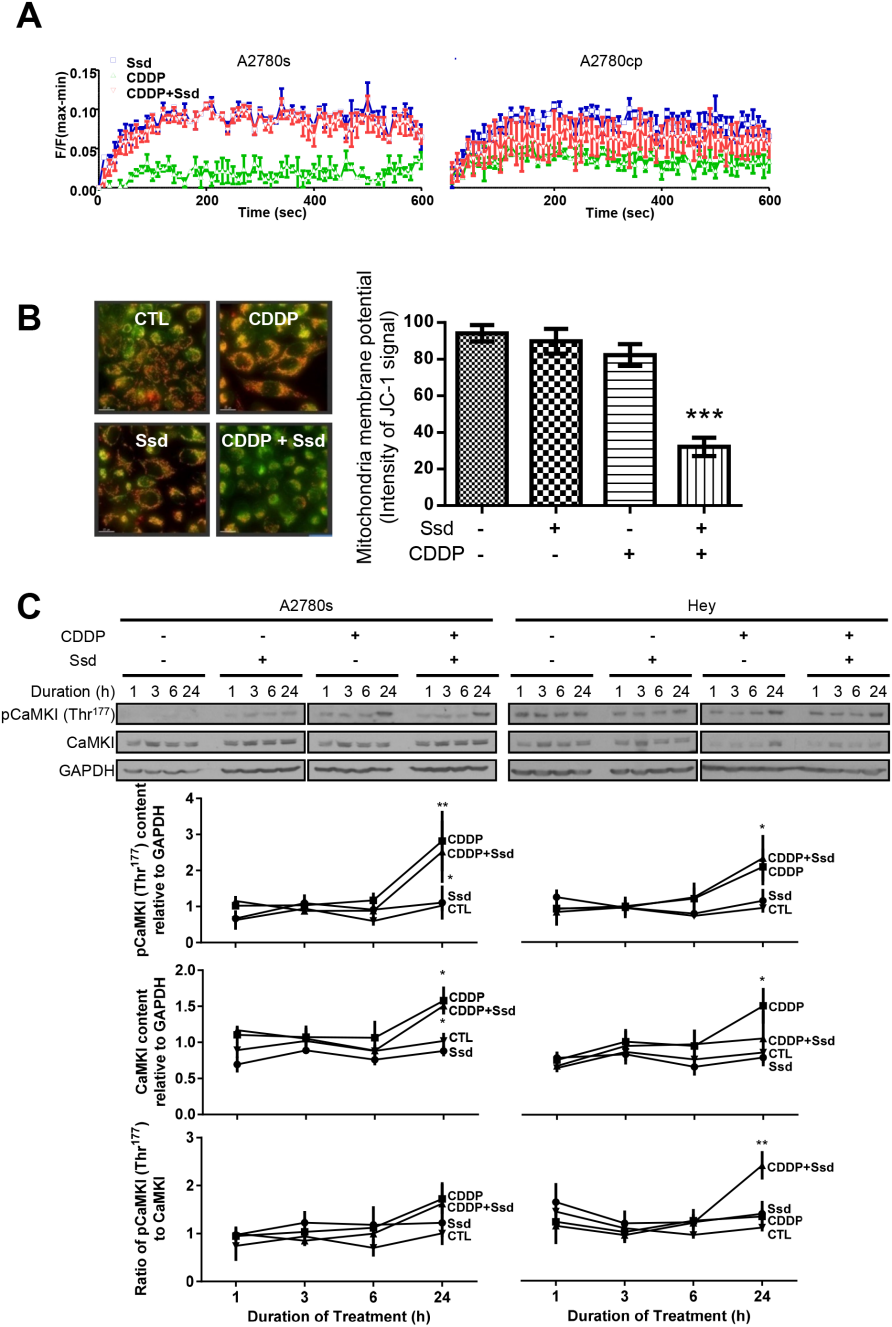
Ssd-increased [Ca2^+^]c and subsequent MMP loss and activation of CaMKI in chemoresistant OVCA cells **(A)** Ssd increased [Ca^2+^]c in OVCA cells, but not CDDP. A2780s and A2780cp cells were cultured with CDDP (10 μM) and/or Ssd (2 μM) and [Ca^2+^]c was measured using the FLIPR Calcium 6 Assay Kit. **(B)** CDDP with Ssd caused MMP loss. A2780cp cells were cultured with CDDP (10 μM) and/or Ssd (2 μM, 24 h) and immunostained with JC-1. **(C)** CDDP in the presence of Ssd increased phospho-Thr^177^-CaMKI contents and the ratio of phospho-Thr^177^-CaMKI to CaMKI. A2780s and Hey cells were cultured with CDDP (10 μM) and/or Ssd (1 μM, 0-24 h). CaMKI, phospho-Thr^177^-CaMKI and GAPDH contents were assessed by WB.^*^P<0.05, ^**^P<0.01 and ^***^P<0.001 (vs respective CTL). (*n*=3).

Previous studies have shown that Ssd increases [Ca^2+^]c by direct inhibition of sarcoplasmic/endoplasmic reticulum calcium ATPase (SERCA), leading to apoptosis and autophagy [7]. CDDP can also increase [Ca^2+^]c in some cancer cells, leading to apoptosis [19–22]. In the present study, Ssd increased [Ca^2+^]c in both chemosensitive (A2780s) and chemoresistant (A2780cp) cells (Supplementary Figure 3A) while CDDP alone was ineffective and had no effect on Ssd-induced Ca^2+^ dynamics (Figure 3A). Although Ssd or CDDP alone showed no significant loss in MMP, a significant decrease in MMP was observed in the presence of both CDDP with Ssd (Figure 3B and Supplementary Figure 3B), supporting the concept that Ssd increases [Ca^2+^]c which in turn is directed into mitochondria by CDDP.

Ssd could induce autophagy through the increase of [Ca^2+^]c and the subsequent activation of Ca^2+^/calmodulin-dependent kinase kinase β (CaMKKβ)–adenosine monophosphate-activated protein kinase (AMPK) pathway [7]. CaMKI is a substrate of CaMKKβ and is also involved in the induction of mitochondrial fragmentation via the activation of Drp1 [13, 14]. Irrespective of the presence of Ssd, CDDP increased the contents of both phospho-Thr^177^-CaMKI and CaMKI, but not the phospho-Thr^177^-CaMKI/CaMKI ratio in chemosensitive cells (A2780s). Ssd alone was ineffective, suggesting CaMKI-Drp1 pathway may be less involved in CDDP-induced mitochondrial fragmentation than Opa1 processing. In contrast, while CDDP alone appeared to have no effect on the contents of phospho-Thr^177^-CaMKI and the phospho-Thr^177^-CaMKI/CaMKI ratio in chemoresistant cells, its action was facilitated by the presence of Ssd, suggesting Ssd may sensitize CDDP-induced CaMKI phosphorylation in chemoresistant cells (Figure 3C). In summary, the increase of [Ca^2+^]c by Ssd can induce the MMP loss and phosphorylation of CaMKI in the presence of CDDP, resulting in Drp1-mediated mitochondrial fragmentation in chemoresistant cells.

### Ssd, in combination with CDDP, decreases mitosis and increases subsequent apoptosis via inhibition of Cdk1 and Cyclin B1 complex in OVCA cells

**Figure 4:**
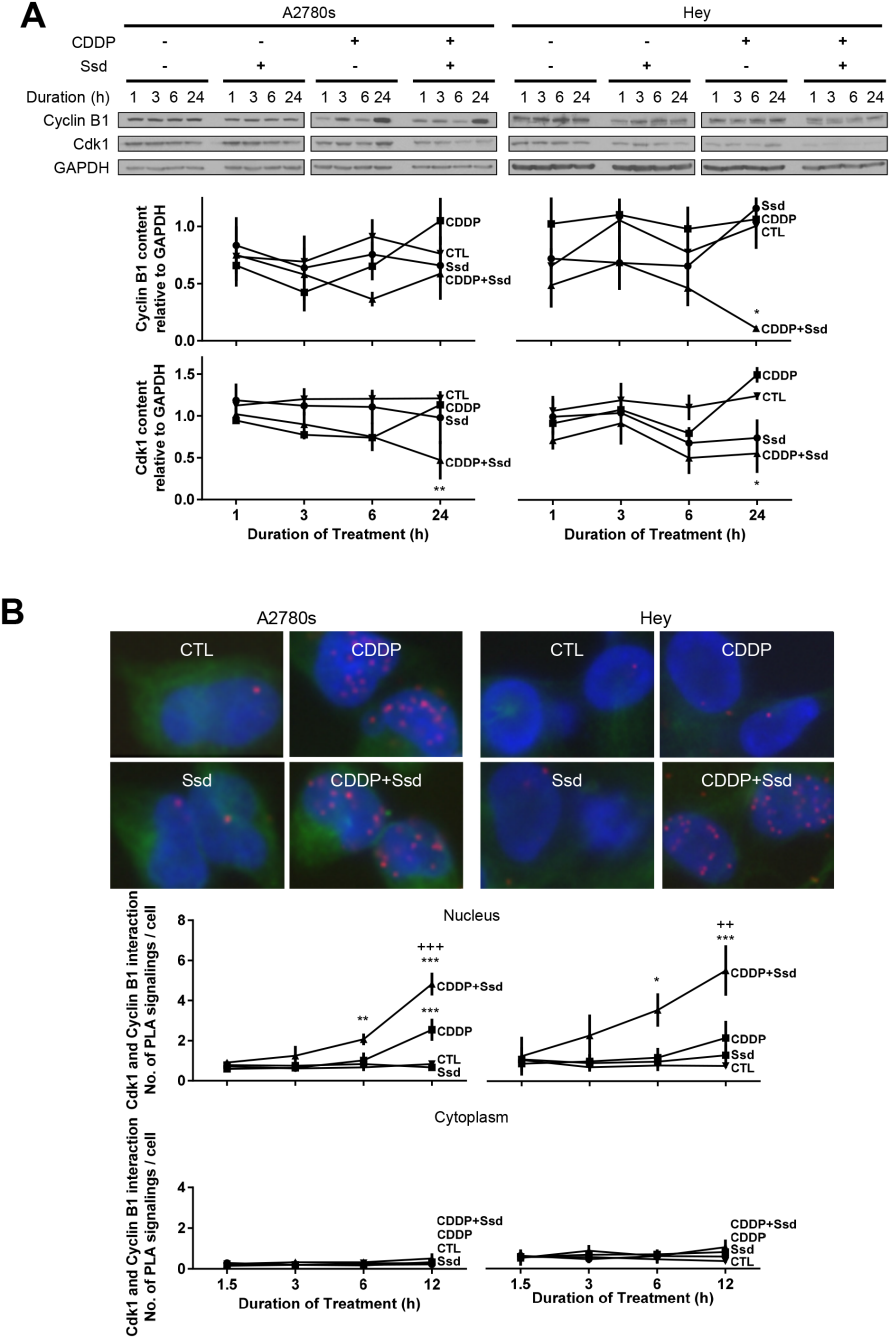
Ssd, in combination with CDDP, decreases mitosis and increases subsequent apoptosis via inhibition of Cdk1 and Cyclin B1 complex in OVCA cells **(A)** CDDP with Ssd decreased Cdk1 and CyclinB1 contents. A2780s and Hey cells were cultured with CDDP (10μM) and/or Ssd (1μM, 0-24h). Cyclin B1, Cdk1 and GAPDH contents were assessed by WB. **(B)** CDDP with Ssd increased the interaction between Cdk1 and Cyclin B1 in chemoresistant OVCA cells. A2780s and Hey cells were cultured with CDDP (10 μM) and Ssd (1 μM, 0-12h) and the interaction between Cdk1 and Cyclin B1 was determined by PLA. ^*^P<0.05, ^**^P<0.01 and^***^P<0.001 (vs respective CTL) and ^++^*P*<0.01 and ^+++^*P*<0.001 (vs respective CDDP). (*n*=3).

In addition to Drp1-mediated mitochondrial dynamics, the Cdk1/Cyclin B1 complex is a major protein kinase involved in mitosis [10]. We demonstrated that CDDP with Ssd significantly decreased the contents of Cdk1 and Cyclin B1 in chemoresistant cells while the combination also significantly decreased the contents of Cdk1 in chemosensitive cells (Figure 4A). Moreover, the combination increased the inactive contents of phospho-Tyr^15^-Cdk1 in cytoplasm in both chemosensitive and chemoresistant cells (Supplementary Figure 4A) while Ssd or CDDP alone was ineffective, suggesting this combination may inhibit Cdk1/Cyclin B1 complex and the translocation of this complex to nucleus. We then determined whether inhibition of Cdk1/Cyclin B1 complex in OVCA cells by Ssd (alone or in combination with CDDP) could lead to G2/M arrest and apoptosis by examining the changes in the amount of mitotic, non-mitotic and apoptotic cells. CDDP increased apoptosis and decreased mitosis in chemosensitive cells, both responses were further enhanced by the presence of Ssd. Although CDDP or Ssd alone elicited minimal or no apoptotic and mitotic response in chemoresistant cells, the responses to CDDP were markedly enhanced by the presence of Ssd (Supplementary Figure 5). In apoptotic cells, the signal of Cdk1 and Cyclin B1 were much weaker than in the cells at interphase, suggesting that Ssd could sensitize chemoresistant cells to CDDP by inhibiting the Cdk1/Cyclin B1 complex, leading to G2/M arrest and subsequent apoptosis. This notion was supported by the observation that while CDDP alone increased the Cdk1/Cyclin B1 interaction in the nucleus, but not in the cytoplasm in chemosensitive cells (A2780s; as determined by in Situ Proximity Ligation Assay (PLA)) and this response was significant enhanced by the presence of Ssd. While CDDP and Ssd alone failed to influence nuclear Cdk1/Cyclin B1 interaction in chemoresistant cells (Hey), significant up-regulation of this response was evident as early as 6 h in the presence of both agonists (Figure 4B).

Although CDDP but not Ssd alone increased the cytoplasmic levels of phospho-Tyr^15^-Cdk1 (inactive form of Cdk1) in chemosensitive but not chemoresistant cells, this response was markedly enhanced in both cell lines in the presence of both CDDP and Ssd (Supplementary Figure 4B). Thus, together CDDP and Ssd decreased the protein contents of Cdk1 and Cyclin B1, increased the levels of phospho-Tyr^15^-Cdk1, and induced G2/M arrest and apoptosis.

### Ssd, in combination with CDDP, induces G2/M arrest via inhibition of PPM1D and subsequent phosphorylation of Chk1 and Cdc25c

**Figure 5:**
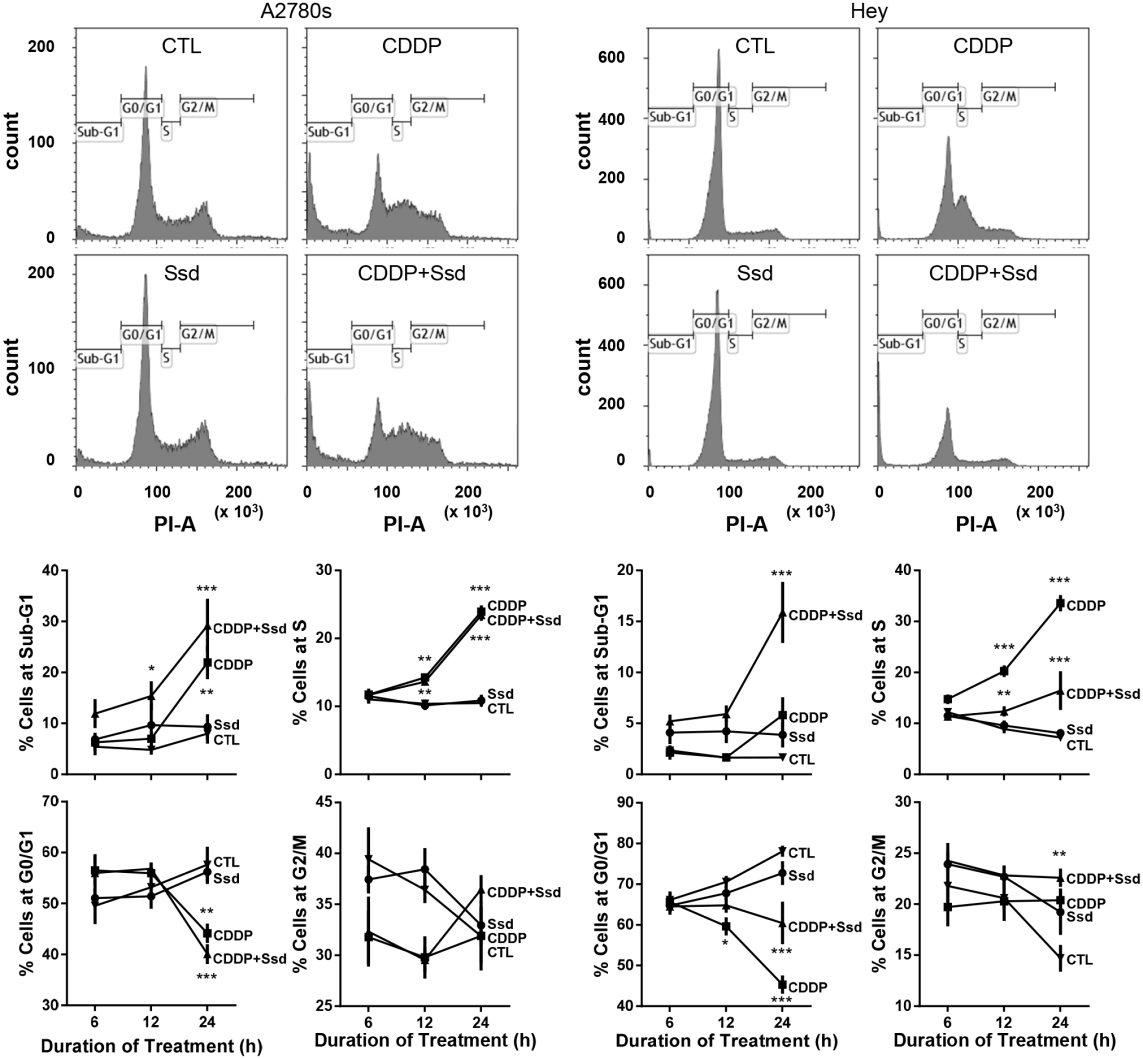
CDDP with Ssd induced G2/M arrest A2780s and Hey cells were cultured with CDDP (10 μM) and/or Ssd (1 μM, 0-24 h), fixed and incubated with RNase A (1 mg/ml) and propidium iodide (25 μg/ml) and analyzed for cell cycle progression using flow cytometer (*n*=3).^*^P<0.05, ^**^P<0.01 and^***^P<0.001 (vs respective CTL).

**Figure 6:**
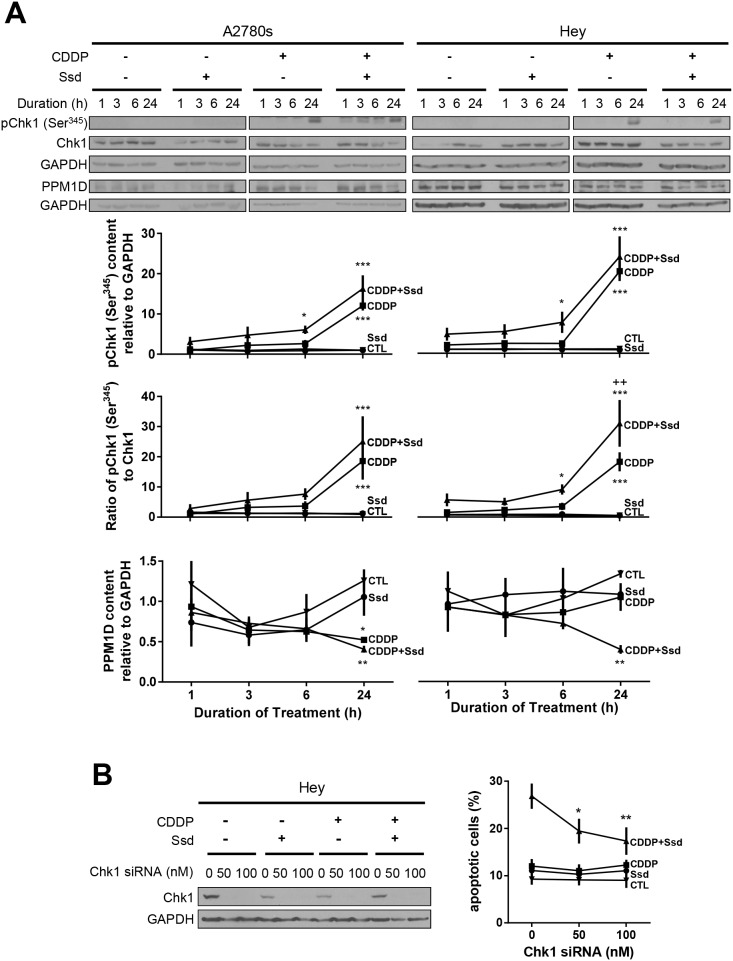
CDDP with Ssd-induced phosphorylation of Ser^345^-Chk1 via decrease of PPM1D, leading to G2/M arrest **(A)** CDDP with Ssd increased phosphor-Ser^345^-Chk1 and decreased PPM1D contents. A2780s and Hey cells were cultured with CDDP (10 μM) and/or Ssd (1 μM, 0-24 h). Phosphor-Ser^345^-Chk1, Chk1, PPM1D and GAPDH contents were assessed by WB (*n*=3).^*^P<0.05, ^**^P<0.01 and^***^P<0.001 (vs respective CTL) and ^++^*P*<0.01 (vs respective CDDP). **(B)** Chk1 siRNA attenuated Ssd-sensitized apoptosis. Hey cells were treated with Chk1 siRNA (0–100 nM, 12 h), cultured with CDDP (10 μM) and/or Ssd (1 μM, 24 h) and apoptosis were assessed (*n*=3). ^*^P<0.05 and ^**^P<0.01 (vs respective siRNA = 0 nM).

To establish the influence of CDDP and Ssd in cell cycle regulation, we examined the upstream kinases (Chk1 and Cdc25c) and phosphatase (PPM1D) as well as G2/M transition known to be involved in the regulation of chemosensitivity in OVCA cells [17]. Irrespective of the presence of Ssd, CDDP decreased G0/G1 and increased Sub-G1 and S phase population, but not G2/M in chemosensitive cells (A2780s) while Ssd alone showed no significant effect (Figure 5). CDDP alone, but not Ssd alone also increased phospho-Ser^216^-Cdc25c (Supplementary Figure 6), phospho-Ser^345^-Chk1 contents and the phospho-Ser^345^-Chk1/Chk1 ratio and decreased PPM1D contents regardless of the presence of Ssd (Figure 6A), suggesting CDDP-induced apoptosis could result from the induction of G1/S arrest and could partly involve the induction of G2/M arrest. On the other hand, in chemoresistant cells (Hey), CDDP alone decreased G0/G1 and increased S phase population, but not sub-G1 and G2/M phase while Ssd alone showed no significant effect (Figure 5). CDDP but not Ssd alone increased phospho-Ser^345^-Chk1 contents and the phospho-Ser^345^-Chk1/Chk1 ratio, but not phospho-Ser^216^-Cdc25c (Supplementary Figure 6) and PPM1D contents (Figure 6A). However, together they significantly increased sub-G1 and G2/M phase population (Figure 5). The combination also increased phospho-Ser^216^-Cdc25c (Supplementary Figure 6) and decreased PPM1D contents (Figure 6A). Moreover, the combination increased phospho-Ser^345^-Chk1 contents and the phospho-Ser^345^-Chk1/Chk1 ratio more than CDDP alone, suggesting CDDP and Ssd together can induce G2/M arrest and apoptosis via inhibition of PPM1D and subsequent phosphorylation of Chk1 and Cdc25c. Apparently, siRNA knockdown of Chk1 attenuated Ssd-sensitized apoptosis in dose-dependent manner (Figure 6B), suggesting the essential role of Chk1 in Ssd-mediated apoptosis.

**Figure 7:**
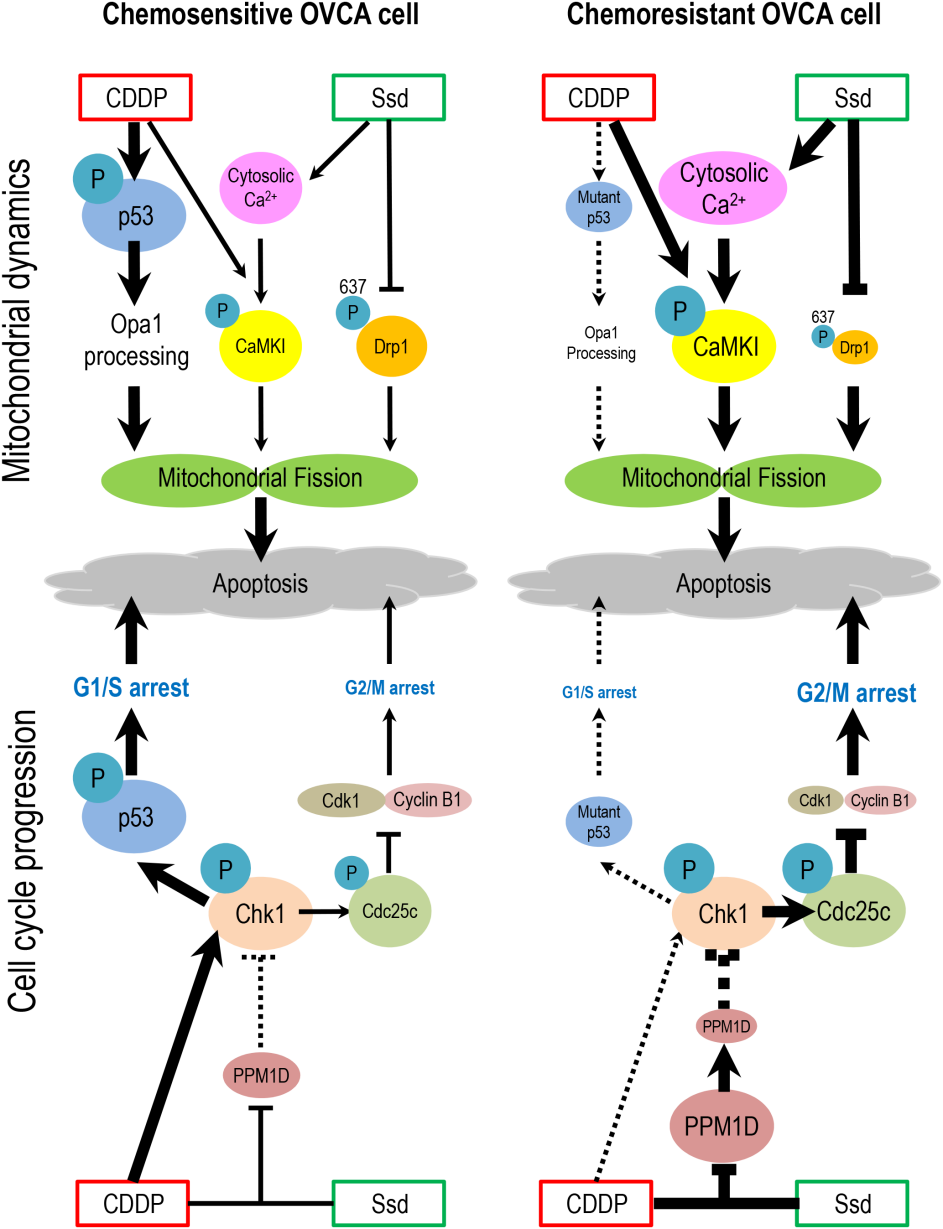
Hypothetical model illustrating the sensitization of chemoresistant OVCA cells by Ssd In chemosensitive cells, CDDP treatment results in the phosphorylation and activation of p53 and activation of Oma1, leading to Opa1 processing, mitochondrial fission and apoptosis. CDDP also activates Chk1, which phosphorylates p53, leading to G1/S arrest and apoptosis. In chemoresistant cells which often exhibit high incidence of p53 mutation and increased PPM1D stability, which inhibits Chk1 activity, CDDP fails to induce G1/S arrest and Opa1 processing. However, Ssd suppresses phospho-Ser^637^-Drp1 content, increases [Ca^2+^]c and, in the presence of CDDP, decreases MMP and increases CaMKI phosphorylation. These actions of Ssd lead to mitochondrial fission and subsequent apoptosis. Moreover, in the presence of CDDP, Ssd decreases PPM1D level, activates Chk1 and increases phospho-Cdc25c content, resulting in G2/M arrest and apoptosis.

## DISCUSSION

In the present study, we demonstrated that Ssd sensitized chemoresistant OVCA cells to CDDP irrespective of their p53 status through induction of mitochondrial fragmentation and G2/M arrest. Mechanistic studies indicate that the action of Ssd involves the activation of calcium signaling, MMP loss and the regulation of proteins controlling in mitochondrial dynamics as well as the inhibition of PPM1D and G2/M check point kinases, respectively. Our findings suggest that Ssd may be a potential novel therapeutic candidate for the sensitization of chemoresistant OVCA cells to CDDP.

Although CDDP and its derivatives are widely used as the first line treatment of OVCA, chemoresistance remains a major therapeutic problem and the molecular basis is still poorly understood. CDDP-induced apoptosis in OVCA cells is dependent on p53 activation [23]. However, a high incidence of p53 mutations are evident in advanced OVCA, especially in high grade serous cancer, leading to the poor prognosis [2]. The treatment strategy with p53-independent manner can overcome their chemoresistance and improve the prognosis of these patients. In the present studies, Ssd exhibited the anti-cancer activities and sensitized chemosensitive and chemoresistant OVCA cells to CDDP irrespective of their p53 status, a notion supported by our p53 gain- and loss-in-function studies. Although Ssd has been reported to induce apoptosis through various mechanisms, including the sensitizing TNF-α-induced cell death via suppressing NF-κB activation or cellular reactive oxygen species (ROS) accumulation in different cancer cell lines, its p53 dependence remains controversial [3–8, 24–26]. The mechanism of Ssd-induced apoptosis in OVCA cells also has not been well studied. In the present studies, we provide new insight into the mechanism of CDDP chemoresistance in OVCA and how Ssd could be useful in their sensitization regardless of p53 status.

Drp1-dependent mitochondrial dynamics in cancer cells may mediate chemosensitivity or confer resistance and appears to be cell type-specific [27]. Drp1 induces mitochondrial fission by its phosphorylation while fusion is mediated by Opa1 via Oma1 activation, but the role of these proteins in chemoresistance is unclear. Here, we explained that depending on the chemosensitivity of the OVCA cells, the induction of mitochondrial fragmentation appeared to be mediated through different regulatory pathways. In contrast to chemoresistant cells, CDDP appeared to induce mitochondrial fragmentation in chemosensitive OVCA cells through activation of Oma1 and p53 and Opa1 processing without detectable influence on phospho-Ser^637^-Drp1 content, a response supported by previous studies [15]. Ssd induced mitochondrial fragmentation in chemoresistant cells via inhibition of phospho-Ser^637^-Drp1 and in the presence of CDDP, induction of Opa1 processing in Oma1-independent manner. We have also demonstrated that hyper-fused mitochondrial morphology is related to cell survival and chemoresistance whereas mitochondrial fragmentation is associated with apoptosis. In the chemoresistant cells, Ssd but not CDDP induced mitochondrial fragmentation, a response associated with decreased phospho-Ser^637^-Drp1 content and augmented by the presence of CDDP. These responses were in part attenuated by mdivi1, suggesting that, in addition to Drp1, other pathway(s) including Opa1 processing could be involved in Ssd-sensitized mitochondrial fragmentation and apoptosis. Our findings are consistent with the observation that chemoresistant OVCA cells exhibit more extensive mitochondrial fusion than their chemosensitive counterparts [15] and induction of apoptosis in gynecologic cancer cells is associated decreased phospho-Ser^637^-Drp1 content and increased mitochondrial fission, both responses being attenuated by mdivi-1 [28, 29]. However, it has also been reported that Drp1 overexpression and mitochondrial fission are associated with CDDP resistance in lung cancer cells [30] and mdivi-1 sensitizes chemoresistant OVCA cells to CDDP [31]. Whether these apparent differences are due to cell-specific responses remains to be determined, personalized therapeutic strategies based on Drp1 status should be considered to overcome the chemoresistance. The role of Oma1 in Opa1 processing in OVCA cells is not clear, since Ssd sensitized CDDP-induced Opa1 processing but failed to influence Oma1 content, suggesting a possible involvement of other proteases in the processing and activation of Opa1 [32].

Ssd also induced mitochondrial fragmentation via MMP loss and activation of CaMKI through Ca^2+^ signaling. Calcium homeostasis is involved in cell proliferation and apoptosis and its dysregulation is believed to be associated with CDDP resistance in various cancer cells [19–22]. Ssd can inhibit SERCA and increase [Ca^2+^]c and apoptosis [7]. In present studies, Ssd significantly increased [Ca^2+^]c in both chemosensitive and chemoresistant OVCA cells while CDDP alone was ineffective and had no effect on Ssd-induced Ca^2+^ dynamics, suggesting that the increase of [Ca^2+^]c may be less involved in CDDP-induced apoptosis in chemosensitive OVCA cells. Ssd or CDDP alone failed to alter MMP, a response significantly decreased when they were present together, supporting the notion that Ssd increased [Ca^2+^]c and, in the presence of CDDP, promoted the entry of Ca^2+^ into mitochondria and MMP loss and mitochondrial fragmentation. It is possible that Ssd can activate CaMKKβ–AMPK signaling pathway which regulates cancer cell fate [7, 33]. CaMKKβ is known to phosphorylate and activate CaMKI (Thr177), which in turn phosphorylates Drp1, leading to mitochondrial fission in various cell types [13, 14]. In chemoresistant cells, CDDP increased the contents of CaMKI, but not of phospho-Thr^177^-CaMKI. However, the presence of Ssd increased phospho-Thr^177^-CaMKI content and the phospho-Thr^177^-CaMKI/CaMKI ratio, supporting the notion that both CDDP and Ssd are required to activate CaMKI and that Ssd increases [Ca^2+^]c and, in the presence of CDDP which could be essential to these pathways as an adjuvant, induces MMP loss and activation of CaMKI, resulting in Drp1-mediated mitochondrial fragmentation and apoptosis in chemoresistant cells. Collectively, CDDP failed to induce Opa1 processing in chemoresistant cells, presumably due to p53 mutation. Instead, Ssd activated Drp1-dependent pathways, including p53-independent, Ca^2+^-mediated down-regulation of phospho-Ser^637^-Drp1 and increased phospho-Thr^177^-CaMKI content, and induced mitochondrial fragmentation, a response enhanced by the presence of CDDP.

PPM1D is overexpressed in chemoresistant OVCA cells and inhibits the action of p53 and Chk1 [17, 18], leading to the inhibition of Chk1-Cdc25c pathway and promotion of mitosis and cell cycle progression. Our studies extended these findings and demonstrated that CDDP and Ssd together inhibited PPM1D, a response accompanied by increased phosphorylation of Chk1, Cdc25c and Cdk1, and G2/M arrest and apoptosis. CDDP treatment increased phospho-Chk1 and phospho-p53 contents and resulted in G1/S arrest [17, 34]. At very high concentrations, Ssd induced G0/G1 arrest in various cancer cells [5, 24, 26], although its influence at lower concentration on cell cycle progression in OVCA is unknown. The mitotic kinase Cdk1/Cyclin B1 is involved in regulation of G2/M transition and their overexpression is predictive of poor survival and chemoresistance in human cancers. Therefore, targeting these proteins could be a new strategy in advanced or chemoresistant cancers [35–39]. In our studies, CDDP and Ssd together inhibited Cdk1/Cyclin B1 complex via the inhibition of PPM1D and subsequently the increase of the phospho-Chk1 and -Cdc25c, suggesting that this combination can overcome the chemoresistance in OVCA cells with p53 mutation via the two different pathways, including the induction of G2/M arrest as well as mitochondrial fragmentation.

In conclusion, we hereby provide new insight into the mechanism of action of Ssd on OVCA cells and demonstrated that Ssd promotes apoptosis and sensitizes chemoresistant OVCA cells to CDDP by facilitating mitochondrial fission and suppressing G2/M transition. In our proposed hypothetical model (Figure 8), in chemoresistant cells which often exhibit high incidence of p53 mutation and increased PPM1D stability, which inhibits Chk1 activity, CDDP fails to induce G1/S arrest and Opa1 processing. However, Ssd suppresses phospho-Ser^637^-Drp1 content, increases [Ca^2+^]c and, in the presence of CDDP, decreases MMP and increases CaMKI phosphorylation. These actions of Ssd lead to mitochondrial fission and subsequent apoptosis. Moreover, in the presence of CDDP, Ssd decreases PPM1D level, activates Chk1 and increases phospho-Cdc25c content, resulting in G2/M arrest and apoptosis. These Ssd-induced biochemical changes are consistent with the notion that Ssd may potentially be important in overcoming the chemoresistance in OVCA.

## MATERIALS AND METHODS

### Reagents

Saikosaponin-d (> 98% purity, HPLC) was purchased from the China Chengdu Biotechnology Company Ltd (Chengdu, China). CDDP (P4349), Hoechst 33258 (94403), and dimethyl sulfoxide (D8418) were from Sigma-Aldrich (St Louis, MO, USA). Roswell Park Memorial Institute (RPMI)-1640 (31800-022), fetal bovine serum (12483-020), penicillin-streptomycin (15140-122), amphotericin B (15290-018), 4’,6-Diamidine-2’-phenylindole dihydrochloride (DAPI) (S36938), Control siRNA (AM4611), Propidium iodide (P3566), Lipofectamine 2000 (11668-019), RNase A (12091021) and N, N, N’, N’-tetramethyl-ethane-1,2-diamine (TEMED) (15524-010) were from Thermo Fisher Scientific (Waltham, MA, USA). Cell lysis buffer (9803S) and Chk1 siRNA constructs (6241) were from Cell Signaling Technology (Danvers, MA, USA). p53 siRNA constructs (sc-29435) was from Santa Cruz Biotechnology (Santa Cruz, CA, USA). Complete Mini Protease inhibitor cocktail tablets (04693159001) and PhosStop phosphatase inhibitor cocktail tablets (04906845001) were from Roche Applied Sciences (Penzberg, Germany). Mdivi1 (3982) was from Tocris Bioscience (Bristol, United Kingdom). Wt-p53 and LacZ adenovirus were synthesized at the University of Ottawa Adenovirus Core Facility (Ottawa, ON, Canada). Minimum Essential Media (MEM), tetraethylbenzimi-dazolylcarbocyanine iodide (JC)-1 dye and Fluo-3- acetoxymethyl (AM) were from Invitrogen (Paisley, Scotland, UK). Antibodies used in the present studies are shown in Supplementary Table 1.

### Cell lines and culture

CDDP-sensitive [A2780s (wt-p53)] and -resistant [A2780cp (p53-mutant), Hey (wt-p53) and SKOV3 (p53-null)] human OVCA cell lines, gifts from Drs Rakesh Goel and Barbara Vanderhyden (Ottawa Regional Cancer Center, Ottawa, ON, Canada), were cultured as previously reported [40, 41]. CDDP-sensitive [SGC-7901] and -resistant [SGC-7901cp] gastric cancer cells (KeyGEN BioTECH, China) were cultured in RPMI-1640 medium. A2780s cells and their resistant counterparts A2780cp cells are epithelial OVCA cells. Hey cells are from moderately differentiated OVCA. SKOV3 cells are of clear cell carcinoma origin. The cells with the different p53-status (A2780s, A2780cp, Hey and SKOV3 cells) were used to explain that Ssd sensitized chemoresistant OVCA cells to CDDP regardless of p53-status. The same p53-status chemosesitive A2780s and chemoresistant Hey cells were used to explain the mechanisms of Ssd-induced sensitization.

### Adenoviral infection

SKOV3 cells were infected with adenoviral wt-p53 (multiplicity of infection (MOI) = 10, 5 h) or LacZ (as control) as previously described [40, 41].

### Apoptosis assay

Apoptosis was determined morphologically using the Hoechst 33258 nuclear stain, as previously described [40, 41]. At least 300 cells were assessed per experimental group.

### Cell viability assay

Cell viability was determined using the 3-(4,5-di-methylthiazol-2-yl)-2,5-diphenyltetrazolium bromide (MTT) and cell viability assays [3].

### Assessment of mitochondrial phenotypes

Assessment of mitochondrial morphology was carried out as previously described [15]. Cells were seeded in 6-well plates, trypsinized, and washed with phosphate buffered saline and centrifuged (900 x g, 1 min). 100μl of the cell suspension was centrifuged (450 x g, 4 min; Shandon Cytospin 4, Thermo Fisher Scientific) using cytological funnels together with silane-coated glass slides [29]. For immunostaining, cells were fixed in 4% paraformaldehyde (1 h, room temperature (RT)) and blocked with 3 % bovine serum albumin. Mitochondria were incubated with anti-Tom20 antibody and appropriate secondary antibody. Confocal images were obtained on Zeiss LM510 inverted microscope (Carl Zeiss, Jena, Germany) with appropriate argon lasers (488nm) and 40X objective. Mitochondrial phenotype of each cell was categorized as tubular or fragmented, as previously described [29]. A cell with “fragmented mitochondria” was defined as the cell with 8 or more individual mitochondrial fragments that were each 3 μm in length across the longest axis. At least 300 cells were analyzed per experimental group.

### Measurement of [Ca^2+^]c

Changes in [Ca^2+^]c were measured by a fluorescent dye, Fluo-3-AM as previously described [42, 43]. A2780s and A2780cp cells were treated with Ssd, cell suspensions were incubated with Fluo-3-AM (5 μM, 37°C, 30 min) and subjected to Fluorescence-activated cell sorting (FACS) analysis (BD Biosciences). At least 10,000 events were analyzed.

### Measurement of cytoplasmic calcium dynamic

Intracellular cytosolic Ca^2+^ dynamic was measured using the FLIPR Calcium 6 Assay Kit (Molecular Devices) according to the manufacturer’s instructions. In brief, A2780s and A2780cp cells were plated at a density of 10,000 cells per well in black wall/clear bottom 96-multiwell plates from Costar (Tewksbury, MA, USA) and cultured for 24 h before treatment. After that, calcium 6 reagent was added directly to cells, and cells were incubated for an additional 2 h at 37 °C and 5% CO2. Ssd and/or CDDP were then added to the wells and immediately subjected to data acquisition on the FLIPR Tetra High-Throughput Cellular Screening System (Molecular Devices) at RT using a 1 s reading interval throughout the experiments.

### MMP detection

Visualization of MMP loss was performed by JC-1 staining. A2780s and A2780cp cells were seeded on confocal disc, treated with Ssd and/or CDDP and stained with JC-1 dye (10 μg/ml, 30 min, 37°C). The retained MMP (Tetramethylrhodamine-isothiocyanate (TRITC) signal) and the loss (Fluoresceinisothiocyanate (FITC) signal) were real-time examined and captured under epifluorescence microscopy (60X objective; Applied Precision DeltaVision Elite, Applied Precision, Inc, USA). At least 500 cells were analyzed per experimental group.

### Protein extraction and western blot analysis

The procedures were performed as previously described [40, 41] and band densities were analyzed (Scion Image software; Scion Corporation, Frederick, MD, USA).

### Immunofluorescence and *in situ* proximity ligation assay (PLA)

Immunofluorescence experiments were performed as previously described [44]. At least 100 cells were analyzed per experimental group.

PLA was conducted according to manufacturer’s instructions (Duolink kit^®^, Sigma-Aldrich). Cells were incubated with a pair of anti-Cyclin B1 and anti-Cdk1antibody, followed by secondary proximity probes (PLA probe PLUS and MINUS). Subsequently, they were incubated with the ligation solution containing ligase and amplification solution containing polymerase and a fluorophore with 594nm excitation and 624 nm emission. Then, cells were incubated with anti-β tubulin antibody and appropriate secondary antibody. Fluorescence signals were detected by Zeiss Axioplan 2 upright fluorescence microscope (40X objective; Carl Zeiss) and analyzed with AxioVision software (Carl Zeiss). PLA-positive signals were quantified using Duolink Image Tool (Sigma-Aldrich). At least 30 cells were analyzed per experimental group.

### Flow cytometry

Cell cycle analysis was performed by flow cytometry, as previously described [17]. A2780s and Hey cells were treated with CDDP and/or Ssd. Cell cycle analysis was performed using BD LSRFortessa Flow Cytometer (BD Biosciences) and data were analyzed with Kaluza Analysis Software (Beckman Coulter Life Sciences, Indianapolis, IN, USA). At least 10,000 events were analyzed per experimental group.

### Statistical analysis

Results are expressed as the mean ± s.e.m. of at least three independent experiments. Statistical assessment was carried out by analysis of variance, using PRISM software (Versions 6.0; GraphPad, San Diego, CA, USA). Differences between experimental groups were determined by the Turkey post-hoc test. Statistical significance was inferred at *P*<0.05.

## SUPPLEMENTARY MATERIALS FIGURES AND TABLE


