# Supplementary material for: Saikosaponin-d, a calcium mobilizing agent, sensitizes chemoresistant ovarian cancer cells to cisplatin-induced apoptosis by facilitating mitochondrial fission and G2/M arrest
Source: Oncotarget. 2017 Sep 16;8(59):99825–40. doi: 10.18632/oncotarget.21076 (PMC5725134; doi:10.18632/oncotarget.21076)
Supplement: Supplementary file 1 [file oncotarget-08-99825-s001.pdf]

Saikosaponin-d, a calcium mobilizing agent, sensitizes chemoresistant ovarian cancer cells to cisplatin-induced apoptosis by facilitating mitochondrial fission and G2/M arrest

SUPPLEMENTARY MATERIALS

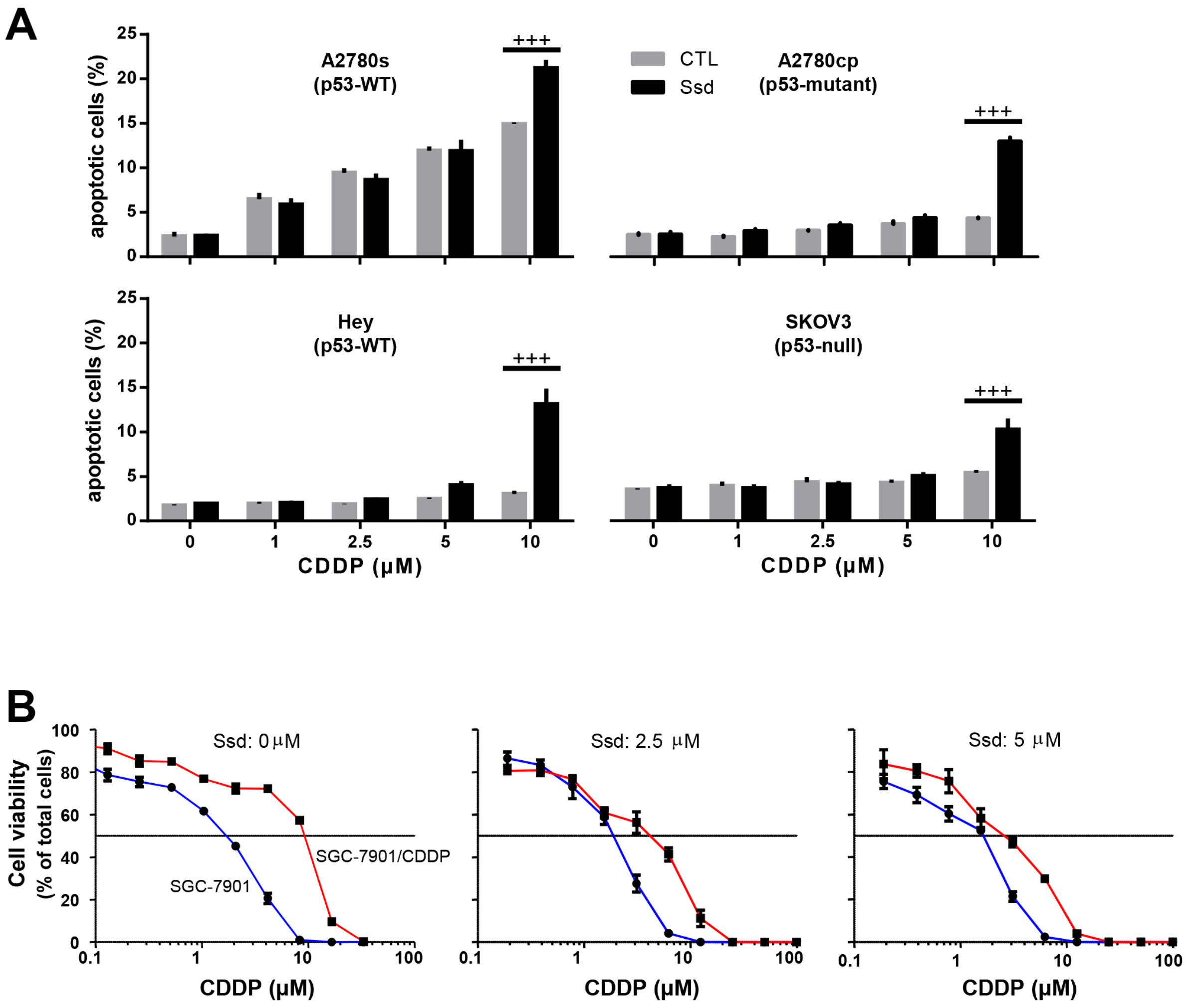

Supplementary Figure 1: Ssd-induced sensitization to CDDP in the different cancer cells. (A) Ssd (1 μM) sensitized OVCA cells to CDDP (10 μM) regardless of p53 status. A2780s, A2780cp, Hey and SKOV3 cells were cultured with CDDP (0-10 μM) and/or Ssd (1 μM, 24 h) and apoptosis were assessed. $^{+++}$*P*<0.001 (vs respective CDDP). **(B)** Ssd-induced sensitization of p53 wild-type gastric cancer cells to CDDP. SGC-7901 cells were cultured with CDDP (0-100 μM) and/or Ssd (0-5 μM, 72 h) and cell viabilities were assessed by MTT assay. (*n*=3).

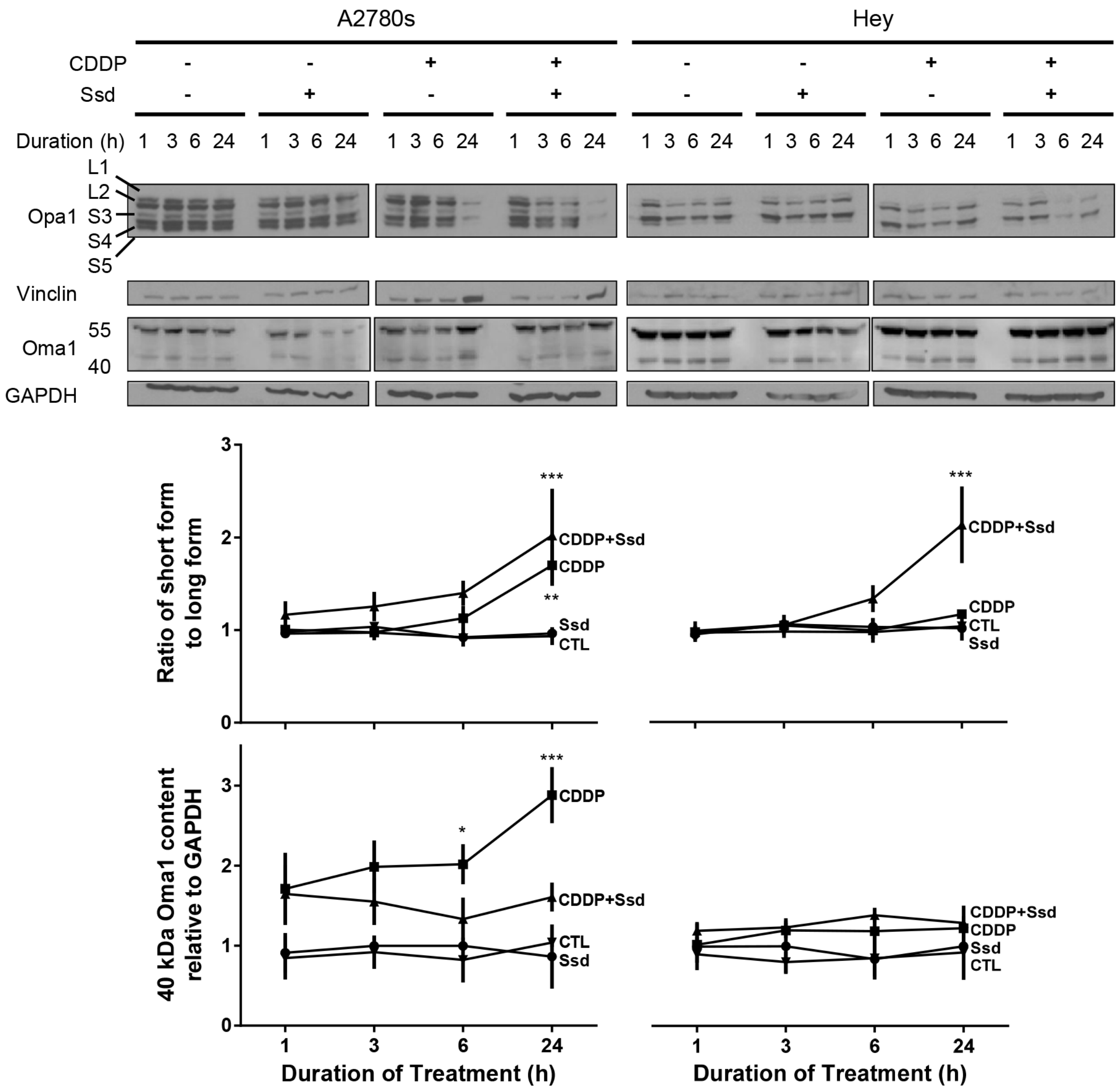

Supplementary Figure 2: CDDP with Ssd-induced Opa1 processing in Oma1 and p53 independent manner. A2780s and Hey cells were cultured with CDDP (10 µM) and/or Ssd (1 µM, 0-24 h). Opa1, Oma1, Vinculin and GAPDH contents were assessed by WB (*n*=3). $^{*}P<0.05$, $^{**}P<0.01$ and $^{***}P<0.001$ (vs respective CTL).

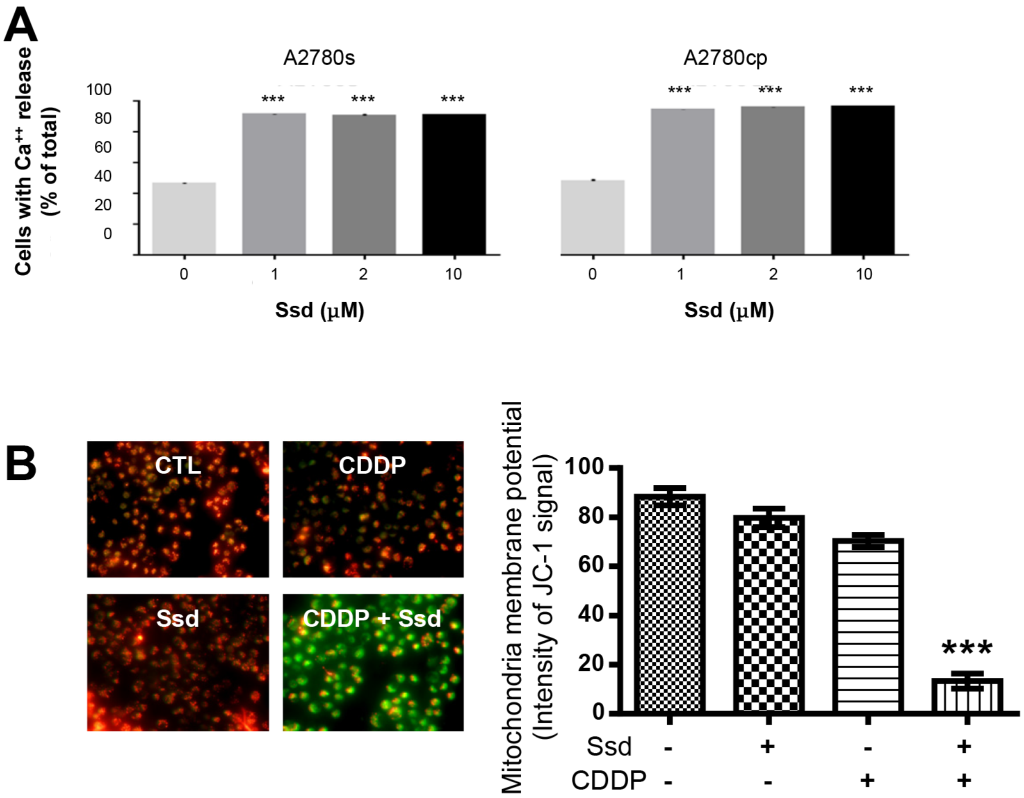

Supplementary Figure 3: Ssd-increased [Ca^{2+}]c and subsequent MMP loss in OVCA cells. (A) A2780s and A2780cp cells were cultured with Ssd (0-10 µM, 4 h), treated with 5 µM Fluo-3-AM and assessed by FACS analysis (*n*=3). $^{***}P<0.001$ (vs respective CTL). **(B)** CDDP with Ssd caused MMP loss. A2780s cells were cultured with CDDP (10 µM) and/or Ssd (2 µM, 24 h) and immunostained with JC-1.

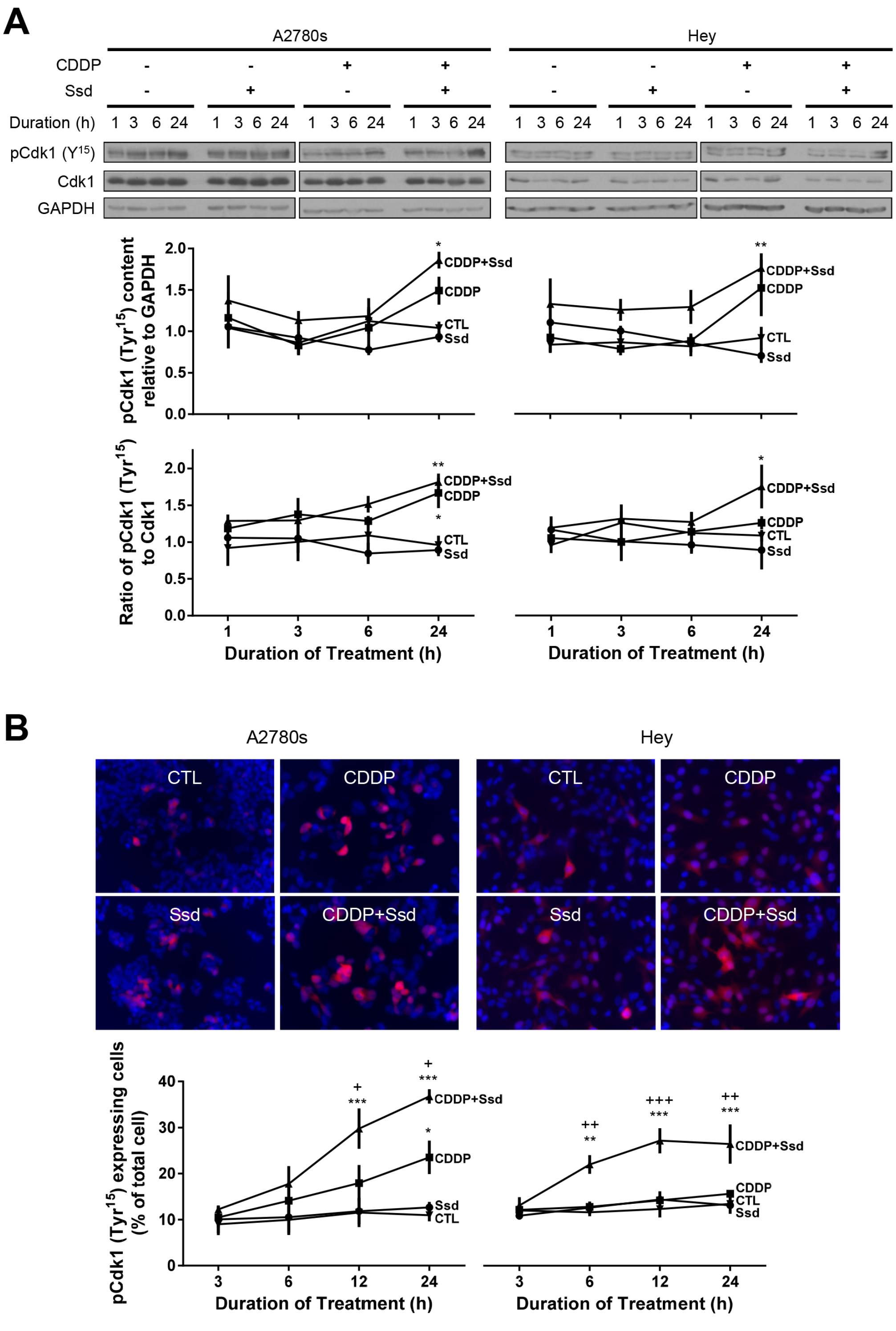

Supplementary Figure 4: CDDP with Ssd-induced phosphorylation of Tyr^{15}-Cdk1 in OVCA cells. **(A)** CDDP with Ssd increased phosphor-Tyr^{15}-Cdk1 contents. A2780s and Hey cells were cultured with CDDP (10 μM) and/or Ssd (1 μM, 0-24 h). Cdk1, phospho-Tyr^{15}-Cdk1 and GAPDH contents were assessed by WB. **(B)** CDDP with Ssd increased the expression of phospho-Tyr^{15}-Cdk1 in cytoplasm after 12 h in chemoresistant cells. A2780s and Hey cells were cultured with CDDP (10 μM) and Ssd (1 μM, 0-24 h) and immunostained with phospho-Tyr^{15}-Cdk1 (n=3). $^{*}P<0.05$, $^{**}P<0.01$ and $^{***}P<0.001$ (vs respective CTL) and $^{+}P<0.05$, $^{++}P<0.01$ and $^{+++}P<0.001$ (vs respective CDDP). (n=3).

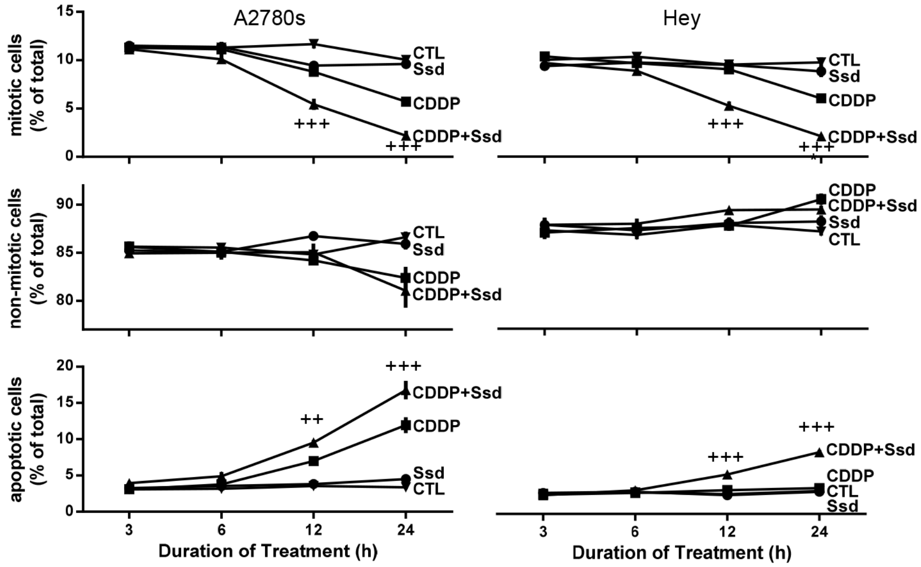

Supplementary Figure 5: CDDP with Ssd increased apoptotic cells and decreased mitotic cells in OVCA cells. A2780s and Hey cells were cultured with CDDP (10 µM) and Ssd (1 µM, 0-24 h), nuclei were immunostained with DAPI and apoptosis and mitosis were assessed (n=3). ^{++}P<0.01 and ^{+++}P<0.001 (vs respective CDDP).

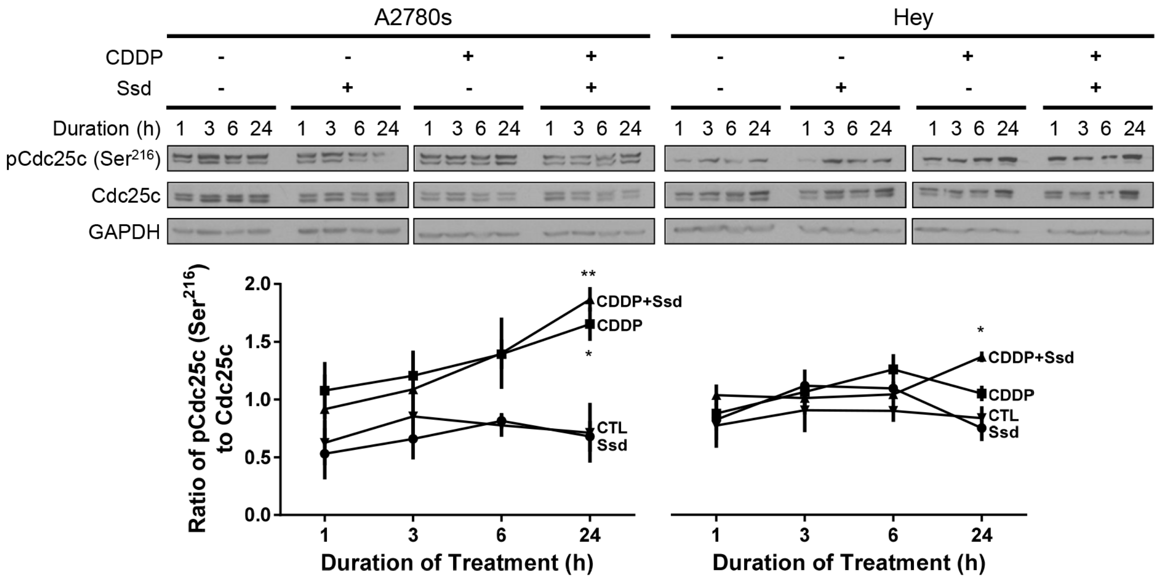

Supplementary Figure 6: CDDP with Ssd-induced phosphorylation of Ser^{216}-Cdc25c. A2780s and Hey cells were cultured with CDDP (10 µM) and/or Ssd (1 µM, 0-24 h). Phospho-Ser^{216}-Cdc25c, Cdc25c and GAPDH contents were assessed by WB (*n*=3). $^{*}P<0.05$ and $^{**}P<0.01$ (vs respective CTL).

Supplementary Table 1: Antibodies used in present studies

A. Primary antibodies

Name	Reactive species	Source	Dilution	company	Catalogue #	Application
phospho-Ser^{345}-Chk1	H, M, R, Mk	Rabbit mAb	1:1000	Cell Signaling Technology	2348	WB
vinculin	H, M, R, Mk	Rabbit pAb	1:1000	Cell Signaling Technology	4650	WB
phospho-Ser^{15}-p53	H, M, R, Mk	Rabbit pAb	1:1000	Cell Signaling Technology	9284	WB
phospho-Ser^{637}-Drp1	R, (H, M, Mk)	Rabbit pAb	1:500	Cell Signaling Technology	4867	WB
Chk1	H, M, R, Mk	Mouse mAb	1:1000	Cell Signaling Technology	2360	WB
phospho-Ser^{216}-Cdc25c	H	Rabbit pAb	1:250	Santa Cruz Biotechnology	sc-12354-R	WB
CaMKI	H, M, R	Mouse mAb	1:100	Santa Cruz Biotechnology	sc-137225	WB
Drp1	H	Mouse mAb	1:250	Santa Cruz Biotechnology	sc-271583	WB
p53	H	Mouse mAb	1:2000	Santa Cruz Biotechnology	sc-98	WB
Cyclin B1	H, M	Mouse mAb	1:100	Santa Cruz Biotechnology	sc-7393	WB
Cdc25c	H	Mouse mAb	1:250	Santa Cruz Biotechnology	sc-13138	WB
Cdk1	H, M, R	Rabbit mAb	1:1000	Abcam	ab32384	WB
phospho-Tyr^{15}-Cdk1	H, M, R	Rabbit mAb	1:1000	Abcam	ab133463	WB
Oma1	H	Rabbit pAb	1:500	Abcam	ab104316	WB
PPM1D	H	Rabbit pAb	1:250	Abcam	ab37495	WB
Cdk1	H, M, R	Mouse mAb	1:1000	Abcam	ab18	WB
GAPDH	H, M, R, Rb, Hm, Mk	Mouse mAb	1:20000	Abcam	ab8245	WB
phospho-Thr^{177}-CaMKI	H, M, R	Rabbit pAb	1:500	LifeSpan BioSciences	LS-C198718	WB
Opa1	H	Mouse mAb	1:1000	BD Biosciences	612606	WB
β Tubulin	H, M, R	Goat pAb	1:100	Santa Cruz Biotechnology	sc-9935	IF
β Tubulin	H, M, R	Rabbit pAb	1:25	Santa Cruz Biotechnology	sc-9104	IF
β Tubulin	H, M, R	Mouse mAb	1:50	Santa Cruz Biotechnology	sc-5274	IF
Tom20	H, M, R	Mouse mAb	1:200	Santa Cruz Biotechnology	sc-17764	IF
Cyclin B1	H, M	Mouse mAb	1:50	Santa Cruz Biotechnology	sc-7393	IF
Cdk1	H, M, R	Rabbit mAb	1:100	Abcam	ab32384	IF
phospho-Tyr^{15}-Cdk1	H, M, R	Rabbit mAb	1:100	Abcam	ab133463	IF

B. Secondary antibodies

Name	dilution	company	Catalogue #	Application
Goat Anti-Rabbit IgG (H + L) horseradish peroxidase conjugate	1:5000	Bio-Rad Laboratories	170-6515	WB
Goat Anti-Mouse IgG (H + L) horseradish peroxidase conjugate	1:5000	Bio-Rad Laboratories	170-6516	WB
Goat Anti-Rabbit Alexa Fluor 555 conjugate	1:400	Thermo Fisher Scientific	A21428	IF
Goat Anti-Mouse Alexa Fluor 488 conjugate	1:400	Thermo Fisher Scientific	A11001	IF
Donkey Anti-Goat Alexa Fluor 488 conjugate	1:400	Thermo Fisher Scientific	A11055	IF

Western Blotting (WB), ImmunoFluorescence (IF), Monoclonal Antibody (mAb), Polyclonal Antibody (pAb) Cell Signaling Technology (Danvers, MA, USA), Santa Cruz Biotechnology (Santa Cruz, CA, USA), Abcam (Cambridge, MA, USA), LifeSpan BioSciences (Seattle, WA, USA), BD Biosciences (San Jose, CA, USA), Thermo Fisher Scientific (Waltham, MA, USA).
